# Interleukin-1β induces CXCR3-mediated chemotaxis to promote umbilical cord mesenchymal stem cell transendothelial migration

**DOI:** 10.1186/s13287-018-1032-9

**Published:** 2018-10-25

**Authors:** Yu-Chien Guo, Yun-Hsuan Chiu, Chie-Pein Chen, Hwai-Shi Wang

**Affiliations:** 1 Institute of Anatomy and Cell Biology, School of Medicine, National Yang Ming University, Peitou, Taipei, 112 Taiwan, Republic of China; 2 Division of High Risk Pregnancy, Mackay Memorial Hospital, Taipei, Taiwan, Republic of China

**Keywords:** Interleukin-1β, Umbilical cord mesenchymal stem cell, CXCR3, CXCL9, Transendothelial migration

## Abstract

**Background:**

Mesenchymal stem cells (MSCs) are known to home to injured and inflamed regions via the bloodstream to assist in tissue regeneration in response to signals of cellular damage. However, the factors and mechanisms that affect their transendothelial migration are still unclear. In this study, the mechanisms involved in interleukin-1β (IL-1β) enhancing the transendothelial migration of MSCs were investigated.

**Methods:**

Immunofluorescence staining and Western blotting were used to observe IL-1β-induced CXC chemokine receptor 3 (CXCR3) expression on MSCs. Quantitative real-time PCR and ELISA were used to demonstrate IL-1β upregulated both chemokine (C-X-C motif) ligand 9 (CXCL9) mRNA and CXCL9 ligand secretion in human umbilical vein endothelial cells (HUVECs). Monolayer co-cultivation, agarose drop chemotaxis, and transwell assay were conducted to investigate the chemotaxis invasion and transendothelial migration ability of IL-1β-induced MSCs in response to CXCL9.

**Results:**

In this study, our immunofluorescence staining showed that IL-1β induces CXCR3 expression on MSCs. This result was confirmed by Western blotting. Following pretreatment with protein synthesis inhibitor cycloheximide, we found that IL-1β induced CXCR3 on the surface of MSCs via protein synthesis pathway. Quantitative real-time PCR and ELISA validated that IL-1β upregulated both CXCL9 mRNA and CXCL9 ligand secretion in HUVECs. In response to CXCL9, chemotaxis invasion and transendothelial migration ability were increased in IL-1β-stimulated MSCs. In addition, we pretreated MSCs with CXCR3 antagonist AMG-487 and p38 MAPK inhibitor SB203580 to confirm CXCR3-CXCL9 interaction and the role of CXCR3 in IL-1β-induced chemotaxis invasion and transendothelial migration.

**Conclusion:**

We found that IL-1β induces the expression of CXCR3 through p38 MAPK signaling and that IL-1β also enhances CXCL9 ligand secretion in HUVECs. These results indicated that IL-1β promotes the transendothelial migration of MSCs through CXCR3-CXCL9 axis. The implication of the finding could enhance the efficacy of MSCs homing to target sites.

**Electronic supplementary material:**

The online version of this article (10.1186/s13287-018-1032-9) contains supplementary material, which is available to authorized users.

## Background

Mesenchymal stem cells (MSCs) are known to home to injured and inflamed regions through the bloodstream to promote wound healing and tissue regeneration [1]. Much is known about leukocyte and lymphocyte chemotactic responses and how these cells migrate with blood circulation to sites of injury and inflamed tissues [2, 3]. Leukocyte homing and migration undergo a series of interactions with endothelial cells (ECs) followed by “transendothelial migration” to enter the target tissue. Several studies have shown that MSCs may possess leukocyte-like, active homing mechanisms that enable them to interact with and migrate across EC monolayers due to injury or inflammation [4, 5]. It is well known that leukocytes can attach and penetrate through ECs, but the interaction between MSCs and ECs and the factors or chemoattractants that affect their transendothelial migration are not well understood. Although MSC homing and migration have been demonstrated, only a small proportion of systemically administered MSCs actually reaches and remains in the target tissue. To overcome these problems, chemokines and their receptors are now recognized as important mediators of stem cell homing.

Injured and inflamed tissue may release cytokines and growth factors such as transforming growth factor (TGF-β1), tumor necrosis factor (TNF-α), and interleukins (IL) [6, 7]. Interleukin-1β (IL-1β) is a member of the interleukin 1 family and plays a key role in innate immunity and inflammation of multiple tissues and organs. Previous studies have found that IL-1β increases bone marrow (BM)-MSC homing and enhances leukocyte migration [8]. However, IL-1β has been found to diminish osteoblast migration [9]. Some studies also indicated that IL-1β induces upregulation of CXC chemokine receptor 3 (CXCR3) in different types of cells, such as T lymphocytes, natural killer cells, and tumor cells [10–14].

CXCR3 is a Gα_i_-protein-coupled seven-transmembrane domain chemokine receptor that plays an important role in various immune responses, transendothelial migration, and metastasis. Previous studies have demonstrated that activated CXCR3 is highly expressed on the surface of effector T cells and is able to regulate T cell trafficking [15, 16]. CXCR3 can specifically bind and respond to three gamma interferon (IFN-γ)-induced CXC chemokine families that are chemokine (C-X-C motif) ligand 9 (CXCL9), CXCL10, and CXCL11. Chemokine ligands and their receptors have been implicated in the pathophysiology of numerous autoimmune and inflammatory diseases and in cancer progression and metastasis. Binding of CXCL9, CXCL10, and CXCL11 to CXCR3 triggers various cellular responses, for example, β1/β2 integrin activation, actin cytoskeleton changes, T cell recruitment, and chemotactic migration [16–19]. Notably, high-level expression of CXCL9 has been shown on some ECs, such as tumor ECs [19] and sclerosis ECs [20]. CXCL9 can be upregulated by TNF-α, IFN-γ, and Toll-like receptors-ligands on human microvascular ECs [21], and CXCL9 can enhance MSC transendothelial migration across murine aortic ECs [22].

MAPKs are a family of serine/threonine kinases and are activated upon their specific substrates at serine and/or threonine residues by a variety of extracellular stimuli including proinflammatory cytokines [23]. A previous study indicated that IL-1β upregulates G protein signaling-4 (RGS4) expression via phosphorylation of p38 MAPK in rabbit colonic smooth muscle cells (SMCs) [24]. Enhancement of CXCL9 expression increases the CXCR3-mediated chemotaxis migration of peripheral blood lymphocytes (PBLs) and T cells through p38 MAPK cascade [25].

To date, the most studied chemokine-chemokine receptor axis in MSC homing to injured and inflamed tissues is CXCR4-SDF-1α. Much research has focused on ways to enhance the expression of CXCR4 on the surface of MSCs to migrate toward chemotactic SDF-1α secreted at injury sites [26, 27]. Our previous studies have found that tail vein-injected human umbilical cord mesenchymal stem cells (hUCMSCs) migrate to the pancreas in hyperglycemic non-obese diabetic (NOD) mice at higher rates than normal NOD mice even in higher levels of interleukin-1β (IL-1β) [28] and CXCL9.

In this study, we found that IL-1β induces chemokine receptor CXCR3 expression on the surface of hUCMSCs through p38 MAPK pathway and confirmed that activated human umbilical vein endothelial cells (HUVECs) can release chemokine CXCL9 ligand that acts as a MSC chemoattractant. IL-1β can also promote and accelerate spreading or transmigrating of MSC on HUVECs. A novel CXCR3 antagonist AMG-487 was used to inhibit CXCR3-mediated chemotaxis migration of IL-1β-treated MSCs. Taken together, this strategy enhances CXCR3-mediated chemotaxis and transendothelial migration abilities for MSC homing to target sites through the CXCR3-CXCL9 axis.

## Methods

### Cell culture

#### Human umbilical cord mesenchymal stem cell (hUCMSC)

Human umbilical cord mesenchymal stem cells were obtained from Bioresource Collection and Research Center, Hsinchu, Taiwan. The culturing conditions were modified from previously described methods [29]. Briefly, MSCs were maintained in a low-serum defined medium consisting of 56% low-glucose Dulbecco’s modified Eagle medium (DMEM; Invitrogen, CA, USA), 37% MCDB 201 (Sigma, MO, USA), 2% fetal bovine serum (Thermo, Logan, UT), 0.5 mg/ml of AlbuMAX® I (Invitrogen, CA, USA), 1× insulin-transferrin-selenium–A (Invitrogen, CA, USA), 1× antibiotic antimycotic solution (Thermo, Logan, UT), 10 nM dexamethasone (Sigma, MO, USA), 50 nM L-ascorbic acid 2-phosphate (Sigma, MO, USA), 10 ng/ml of epidermal growth factor (PeproTech, NJ, USA), and 1 ng/ml of platelet-derived growth factor-BB (PeproTech, NJ, USA) at 37 °C and 5% CO_2_. When cells reached 70–80% confluence, cells were detached using HyQtase (Thermo, Logan, UT) and replated at a ratio of 1:4.

#### Human umbilical vein endothelial cell (HUVEC)

Human umbilical vein endothelial cells were purchased from Bioresource Collection and Research Center, Hsinchu, Taiwan. The culture method was modified from protocol described by Gupta et al. [30]. Briefly, HUVECs were cultured on a 1% gelatin (Sigma, MO, USA)-coated T75 flask, maintained in Dulbecco’s modified Eagle medium/Nutrient Mixture F-12 (Life Technologies, NY, USA) supplemented with 2% fetal bovine serum (Thermo, Logan, UT), 1 μg/ml hydrocortisone (Sigma, MO, USA), 5 ng/ml epidermal growth factor (PeproTech, NJ, USA), 10 ng/ml FGF-2 (PeproTech, NJ, USA), 20 μg/ml heparin sulfate (Sigma, MO, USA), 250 ng/ml insulin (Sigma, MO, USA), and 1× penicillin-streptomycin solution (Thermo, Logan, UT) at 37 °C and 5% CO_2_. When cells reached 80–90% confluence, they were detached using HyQtase (Thermo, Logan, UT) and replated at a ratio of 1:3.

### Cytokines and inhibitors

MSCs and HUVECs were cultured for 12–16 h in DMEM containing 0.5% FBS and 3–4 h in F-12 containing 1% BSA, respectively. In our previous study, MSC migration can be significantly enhanced by adding 100 ng/ml human recombinant interleukin-1β (IL-1β) [31]. In this study, both MSCs and HUVECs were stimulated with 100 ng/ml IL-1β (Peprotech, NJ, USA) for 30 min and 12 h individually. The protein synthesis inhibitor cycloheximide (Cayman, USA) was added to MSCs 60 min prior to IL-1β stimulation at a final concentration of 20 μg/ml. The CXCR3 antagonist (±)-AMG 487 (Tocris, UK) was added to culture MSC medium 2 h prior to stimulation at 500 nM. The p38 MAPK inhibitor SB203580 (Tocris, UK) was added to MSCs 2 h prior to stimulation at concentrations of 25 μM, 10 μM, and 20 μM, respectively.

### MTT cell viability assay

MSCs were cultured for 12–16 h in DMEM containing 0.5% FBS, then the CXCR3 antagonist (±)-AMG 487 (500 nM) and p38 MAPK inhibitor SB203580 (25 μM) were added to MSCs 2 h prior to 30 min of IL-1β (100 ng/ml) stimulation or 24 h of CXCL9 (500 ng/ml) stimulation. HUVECs were cultured for 3–4 h in F-12 containing 1% BSA then stimulated with IL-1β for 12 h or CXCL9 for 24 h. Both MSCs and HUVECs were incubated with 1 mg/ml MTT reagent, 3-(4,5-dimethylthiazol-2-yl)-2,5-diphenyltetrazolium bromide (SERVA Heidelberg German 20395) in culture medium for 4 h, then suspended in DMSO (Sigma, MO, USA) for 2 h at 37 °C. The results were detected at a wavelength of 545 nm using multimode microplate readers (Infinite 200, TECAN).

### Quantitative real-time polymerase chain reaction (qPCR)

After stimulation with IL-1β, cells were harvested and total RNA was extracted using TriPure isolation reagent (Bioline, London, UK) according to the manufacturer’s instruction. RNA was converted to cDNA with the Tetro cDNA Synthesis kit (Bioline, London, UK). The same amount of cDNA (100 ng) was used in each reaction. The oligonucleotides used for each gene are provided in Additional file 1: Table S1. Gene expression was analyzed by quantitative real-time PCR using SensiFAST CYBR Hi-ROX System (Bioline, London, UK) and each reaction was repeated in triplets.

### CXCL9 enzyme-linked immunosorbent assay (ELISA)

After stimulation with IL-1β, the HUVEC condition medium was collected at the time point of 18 h and 24 h. Quantitation of CXCL9 protein expression was performed using Human CXCL9/MIG Quantikine ELISA Kit (R&D systems, USA). The results were detected at a wavelength of 450 nm using multimode microplate readers (Infinite 200, TECAN).

### Cell immunofluorescence and image

Cells were plated in 24-well plates with 12-mm coverslips, cultured for 12–16 h in DMEM containing 0.5% fetal bovine serum and then stimulated 100 ng/ml IL-1β with or without inhibitor pretreatment. Cells were fixed with PBS containing 4% (*v*/*v*) paraformaldehyde for 15 min and permeabilized with 0.1% Triton X-100 in PBS for 15 min. Cells were blocked with 2% BSA (Sigma, MO, USA) in PBS for 1 h and incubated with primary anti-CXCR3 antibody (1:200) (Abcam, London, UK) overnight. Cells were incubated with Alexa Fluor 488 secondary antibody (1:200) for 1 h in room temperature. Cells were then stained with Hoechst 33258 (1:5000) (Sigma, MO, USA) after washing three times with PBS. Samples were mounted with fluorescence mounting medium (Ibidi, Planegg, Germany) and imaged using a fluorescent microscope (DM6000B, Leica) or laser confocal microscope (FV1000, Olympus). The fluorescence intensity was then processed and analyzed using Image J.

### Western blotting

Cells were washed with 1× PBS before the cytosolic and membrane proteins were fractionated by Mem-PER™ Plus Membrane Protein Extraction Kit (Thermo, IL, USA), according to the manufacturer’s instructions. Protein concentration was determined with the Coomassie Plus (Bradford) protein assay reagent (Thermo, IL, USA) using multimode microplate readers (Infinite 200, TECAN). Protein samples were resolved with 10% sodium dodecyl sulfate-polyacrylamide gel electrophoresis (SDS-PAGE) and transferred to polyvinylidene fluoride membranes (Merck, Darmstadt, Germany). Membrane was blocked in 10% fish gelatin blocking buffer (AMRESCO, OH, USA) for 1 h and then incubated with the primary anti-CXCR3 antibody (Abcam, London, UK) at 1:3000 dilution at 4 °C overnight. Pan-cadherin (GeneTex, CA, USA) was used as a cell plasma membrane marker. The blots were washed with Tris-buffered saline with Tween 20 (TBST) and incubated with Rabbit IgG (HRP) (GeneTex, CA, USA) secondary antibody for 1 h at room temperature. Membranes were washed and then detected by enhanced chemiluminescence substrate using Luminescence Imaging System (LAS-4000, GE, USA).

### Monolayer co-cultivation assay

HUVECs were seeded on 0.1% gelatin-coated coverslips in 24-well plates and incubated for 24–48 h until cells formed a confluent monolayer. HUVEC monolayers were stained with 8 μM Calcein AM (Tocris, UK) in serum-free medium for 30 min at 37 °C, then gently washed three times with PBS. Prior to co-cultivation, CellTracker™ Orange (Life Technologies, NY, USA) was added at final concentration of 5 μM to the MSC cell suspension. After incubation for 40 min at 37 °C, MSCs were centrifuged at 800*g* for 2 min, the medium was aspirated, and pellets were washed with PBS three times. For co-cultivation, labeled MSCs were placed on HUVEC monolayers for 30, 60, 180, 240 min. Thereafter, cells were fixed with 4% (*v*/*v*) paraformaldehyde, and morphology and interaction between HUVECs and MSCs in the X-Z and Y-Z plane were analyzed by laser confocal microscope.

### Agarose drop chemotaxis assay

0.1 g of low-melting point agarose powder (Basic Life, USA) was added into 20 ml PBS to prepare 0.5% agarose solution. The solution was heated until boiling and filtered into a 50-ml centrifuge tube immediately. After cooling down to 40 °C, 90 μl of agarose solution was pipetted into an Eppendorf tube containing 10 μl of serum-free medium with or without human CXCL9. Fifty microliters of agarose solution was added as rapidly as possible to gelatin-coated 18-mm coverslips in 6-well plates, and the drops were allowed to solidify at 4 °C. Before agarose drop chemotaxis assay, MSCs were cultured for 12–16 h in DMEM containing 0.5% FBS, then CXCR3 antagonist (±)-AMG 487 (500 nM) and p38 MAPK inhibitor SB203580 (25 μM) were added to MSCs 2 h prior to IL-1β stimulation. For agarose drop chemotaxis assay, MSCs were then detached with HyQtase (Thermo, Logan, UT), and 2 × 10^5^ cells in serum-free DMEM were plated on coverslips with agarose drop. After 24 h incubation at 37 °C, the number of migrated MSCs was counted under complete agarose drop by microscopy.

### In vitro transendothelial migration assay

Transwell inserts with 8-μm pore size were coated with 0.5% gelatin, and 2 × 10^4^ HUVECs were seeded onto the membrane of each transwell insert, incubated over 2 days until forming a HUVEC monolayer and then activated with or without IL-1β for 12 h in serum-free F-12 containing 1% BSA. Before transendothelial migration assay, MSCs were cultured for 12–16 h in DMEM containing 0.5% FBS, then CXCR3 antagonist (±)-AMG 487 (500 nM) and p38 MAPK inhibitor SB203580 (25 μM) were added to MSCs 2 h prior to IL-1β stimulation. MSCs were then detached with HyQtase and stained with CellTracker™ Orange at a concentration of 10 μM. After MSCs were incubated for 40 min at 37 °C, then spun down at 800*g* for 2 min, the medium was aspirated and the pellets were washed with PBS three times. For transendothelial migration assay, 1.5 × 10^4^ labeled MSCs in 200-μl serum-free DMEM were loaded into the upper chamber; meanwhile, 500-μl serum-free F-12 with or without 50 ng/ml human CXCL9 was added to the lower chamber. After 24 h incubation at 37 °C, non-migrated cells in the lower chamber were gently removed with cotton swabs. A number of MSCs which had migrated through to the lower chamber were fixed and stained with Hoechst 33258, and HUVECs were stained with Hoechst 33258 without CellTracker™ Orange to distinguish two types of cells. Fluorescence microscopy was used to count the number of migrated cells in five randomly selected fields.

### Statistical analysis

Statistical analyses were performed using Prism 5 software. Quantitation data were analyzed by Student’s *t* test and one-way ANOVA. *P* values < 0.05 were considered statistically significant.

## Results

### IL-1β induces rapid CXCR3 expression on the surface of MSCs

**Fig. 1:**
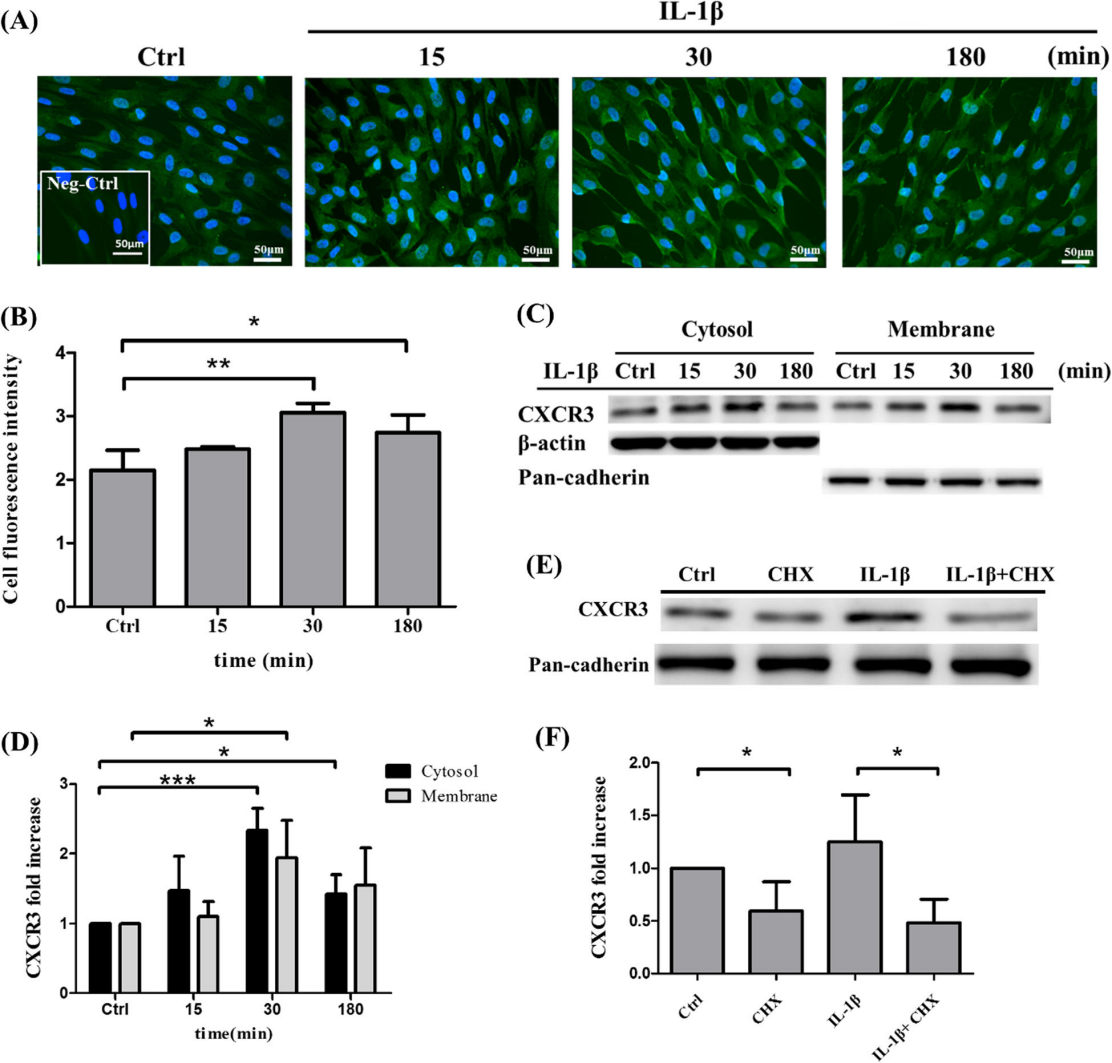
IL-1β induces CXCR3 expression on MSCs. **a** Immunofluorescence staining of CXCR3 (green) in control MSCs and MSCs stimulated with 100 ng/ml IL-1β at 15, 30, and 180 min; cellular nuclei were stained in blue. Negative control: CXCR3 antibody without secondary antibody. Scale bar: 50 μm. **b** Quantitative fluorescence intensity results analyzed by Image J; 10 cells were quantitated in each experiments of control MSCs and MSCs stimulated with 100 ng/ml IL-1β at 15, 30, and 180 min. The data represent mean ± SD (*n* = 3). Statistical analysis was determined by Student’s *t* test and one-way ANOVA. *P < 0.05, **P < 0.01. **c** MSCs were stimulated with 100 ng/ml IL-1β at 15, 30, and 180 min. The cytosolic and membrane protein were fractionated by Mem-PER™ Plus Membrane Protein Extraction Kit, then the expression of CXCR3 (41 kDa) protein was detected by Western blotting. β-actin was used as a cytosolic marker whereas pan-cadherin was used as a cell membrane marker. **d** Quantitative results of the Western blotting of CXCR3 cytosolic and membrane protein expression of **c**. The data represent mean ± SD (*n* = 3). Statistical analysis was determined by Student’s *t* test and one-way ANOVA. *P < 0.05 versus control, ***P < 0.001 versus control. **e** MSCs were pretreated with protein synthesis inhibitor, cycloheximide, at concentration of 20 μg/ml, then stimulated with IL-1β for 30 min. The expression of CXCR3 membrane protein was detected by Western blotting. **f** Quantitative results of the Western blotting of CXCR3 membrane protein expression of **e**. The data represent mean ± SD (*n* = 3). Statistical analysis was determined by Student’s *t* test and one-way ANOVA. *P < 0.05

To determine the location of chemokine receptor CXCR3 after stimulation with 100 ng/ml IL-1β for 15, 30, and 180 min, immunofluorescence staining was performed (Fig. 1a). The staining fluorescence intensity was quantitated (Fig. 1b). The results showed that CXCR3 is an integral membrane protein and can be upregulated on the cell surface of MSCs by IL-1β. In addition, MSCs expressed the highest CXCR3 levels on the surface after 30 min of stimulation in comparison with 15 and 180 min of stimulation. To further confirm whether IL-1β could induce CXCR3 expression on protein levels in MSCs, membrane and cytosolic proteins were fractionated using Mem-PER™ Plus Membrane Protein Extraction Kit and then detected using Western blotting. We found that CXCR3 was upregulated both in cytosolic and membrane proteins compared with control in MSCs after incubation with IL-1β with significant enhancement at 30 min rather than 15 and 180 min (Fig. 1c, d). The cell viability assay indicated no significant change in IL-1β-treated MSCs in comparison to the control group, which suggests that IL-1β-induced CXCR3 expression was not affected by cell proliferation (Additional file 2: Figure S1A). To determine CXCR3 protein production through intracellular translocation or protein synthesis, MSCs were pretreated with cycloheximide for 1 h and analyzed by Western blotting to identify CXCR3 expression. Cycloheximide is a protein synthesis inhibitor and has been shown to block the translation elongation in organisms [32]. The results indicated that the level of CXCR3 protein expression in IL-1β-treated cells was higher than that in non-treated cells. Meanwhile, pretreatment of 20 μg/ml cycloheximide of MSCs significantly suppressed IL-1β-induced CXCR3 protein expression in membrane (Fig. 1e, f). The cell viability assay indicated no significant change in IL-1β, cycloheximide-treated MSCs in comparison to control MSCs. The results show that this concentration of IL-1β and cycloheximide did not lead to cell death (Additional file 2: Figure S1A). We thus demonstrated that the cell surface expression of CXCR3 induced by IL-1β is apparently dependent upon de novo protein synthesis.

### IL-1β upregulates CXCL9 expression in HUVECs

**Fig. 2:**
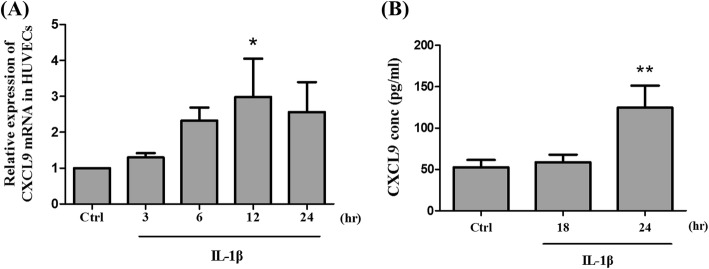
Effects of IL-1β in HUVECs on CXCL9 mRNA and CXCL9 ligand secretion. **a** HUVECs were stimulated with 100 ng/ml IL-1β at the time point of 3, 6, 12, and 24 h. The CXCL9 mRNA expression was detected using quantitative real-time PCR. **b** HUVECs were stimulated with IL-1β then the HUVEC conditioned medium was collected at 18 and 24 h. The CXCL9 ligand protein was detected by ELISA to quantify the release of CXCL9 in supernatants. The data represent mean ± SD (*n* = 3). Statistical analysis was determined by Student’s *t* test and one-way ANOVA. *P < 0.05 versus control, **P < 0.01 versus control

To identify the relevance of CXCL9 expression by IL-1β in HUVEC, SYBR Green quantitative real-time PCR was performed. Figure 2a shows that the level of CXCL9 mRNA transcript in IL-1β-treated cells was higher than that in non-treated cells. CXCL9 mRNA peaked at 12 h fourfold above the control group and then decreased by 24 h. To further evaluate whether HUVECs could produce CXCL9 ligand proteins in response to IL-1β, ELISA assay was used to detect the secretion level of CXCL9 ligand protein in the condition medium of IL-1β-treated HUVECs. After treatment with IL-1β for 24 h, release of extracellular CXCL9 ligand protein was observed (Fig. 2b). These finding were consistent with our quantitative real-time PCR results (Fig. 2a). Thus, we confirmed that IL-1β could upregulate both CXCL9 mRNA and CXCL9 ligand secretion in HUVECs. The cell viability assay indicated no significant change in IL-1β-treated HUVECs in comparison to the control group. The results show that IL-1β-induced HUVEC expression was not affected by cell viability (Additional file 2: Figure S1B).

### Effects of IL-1β in morphology and interaction between HUVECs and MSCs

**Fig. 3:**
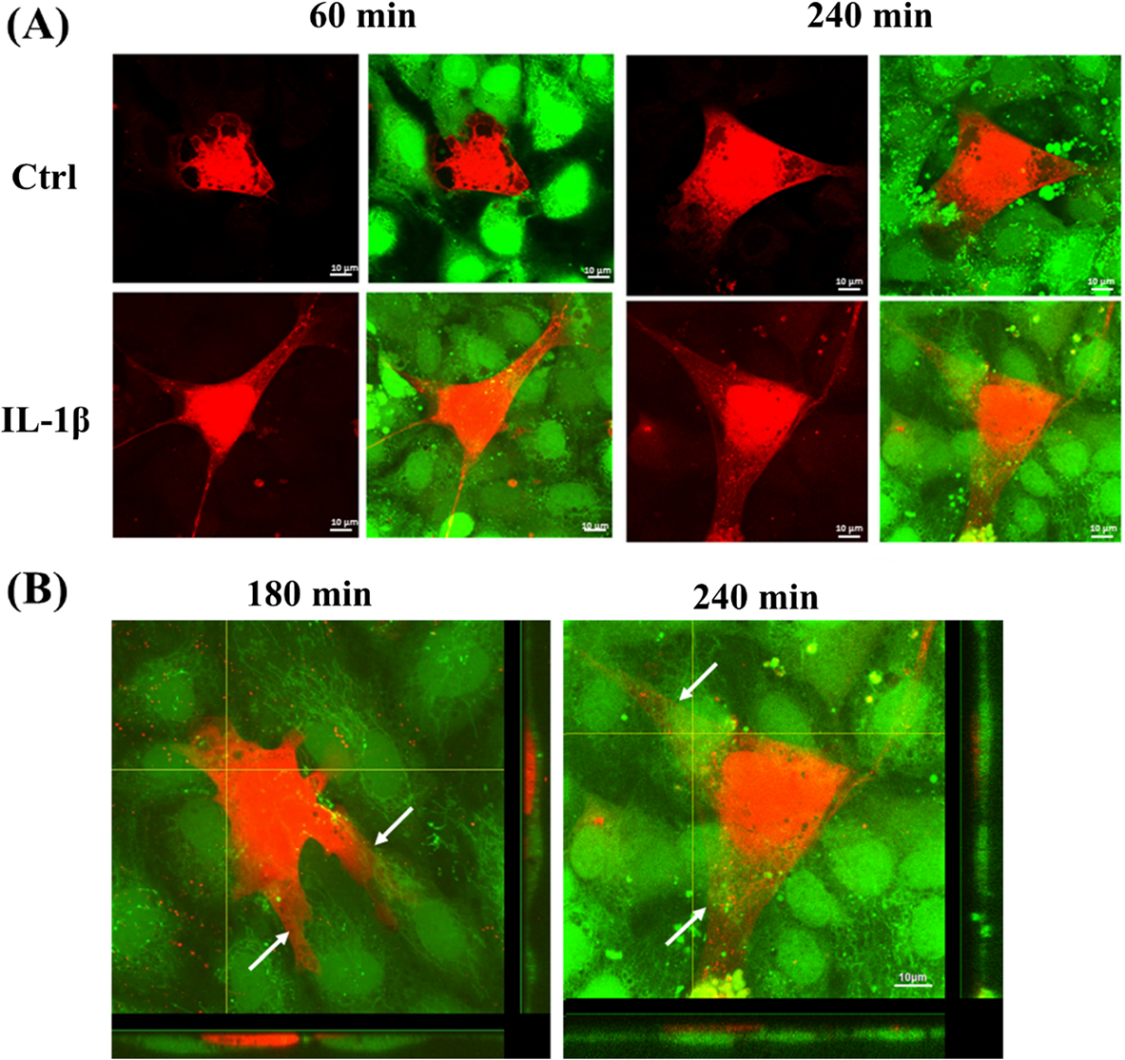
Effects of IL-1β in morphology and interaction between HUVECs and MSCs. Labeled MSCs with CellTracker™ Orange were seeded on HUVECs stained with Calcein AM and co-cultivated for 30 to 240 min. After a period of 60 min, MSCs attached to HUVECs and the morphology were still spherical but developed form of cytoplasmic offshoot. **a** After 60 min, MSC became flattened and adhered to HUVEC monolayer. IL-1β promoted adhesion (left, 60 min) and transendothelial migration abilities (right, 240 min) of MSCs. **b** After 180 and 240 min, MSCs extended long plasmic filopodia and integrated into the HUVEC monolayer. Orthogonal projections illustrate that MSCs inserted into HUVEC monolayer (left, 180 min) and formation of filopodia caused transendothelial migration (right, 240 min). Arrows indicate MSC migration through the HUVEC. Horizontal bar: XZ plane of confocal image stack; vertical bar: YZ plane of confocal image stack. Scale bar = 10 μm

After we finished our investigation of CXCR3 expression on MSCs and CXCL9 ligand secretion in HUVECs, we further investigated the interaction between MSCs and HUVECs by using a co-cultivation approach. As shown in Fig. 3a, b, we observed time-related changes in the morphology of the MSCs. Figure 3a shows that MSCs began to attach to HUVECs and the cell form of cytoplasmic offshoot appeared at the cell edge after 60 min, at which point the MSCs became more flattened and adhered to the HUVEC monolayer. When MSCs were stimulated with IL-1β for 30 min, we found that MSCs attached and adhered to HUVECs in less time. IL-1β-treated MSCs extended filopodia and integrated into the edge of HUVECs compared with control after 60 min (Fig. 3a). Furthermore, IL-1β-treated MSCs could perform faster transendothelial migration than control MSCs after 240 min (Fig. 3a). After IL-1β treatment for 180 min, MSCs extended long plasmic filopodia and integrated into the HUVEC monolayer, and after 240 min, the IL-1β-treated MSCs penetrated and transmigrated underneath HUVECs (Fig. 3b).

### CXCL9 enhances MSC chemotaxis migration via CXCR3-mediated MSC chemotaxis invasion and MSC transendothelial migration induced by IL-1β

**Fig. 4:**
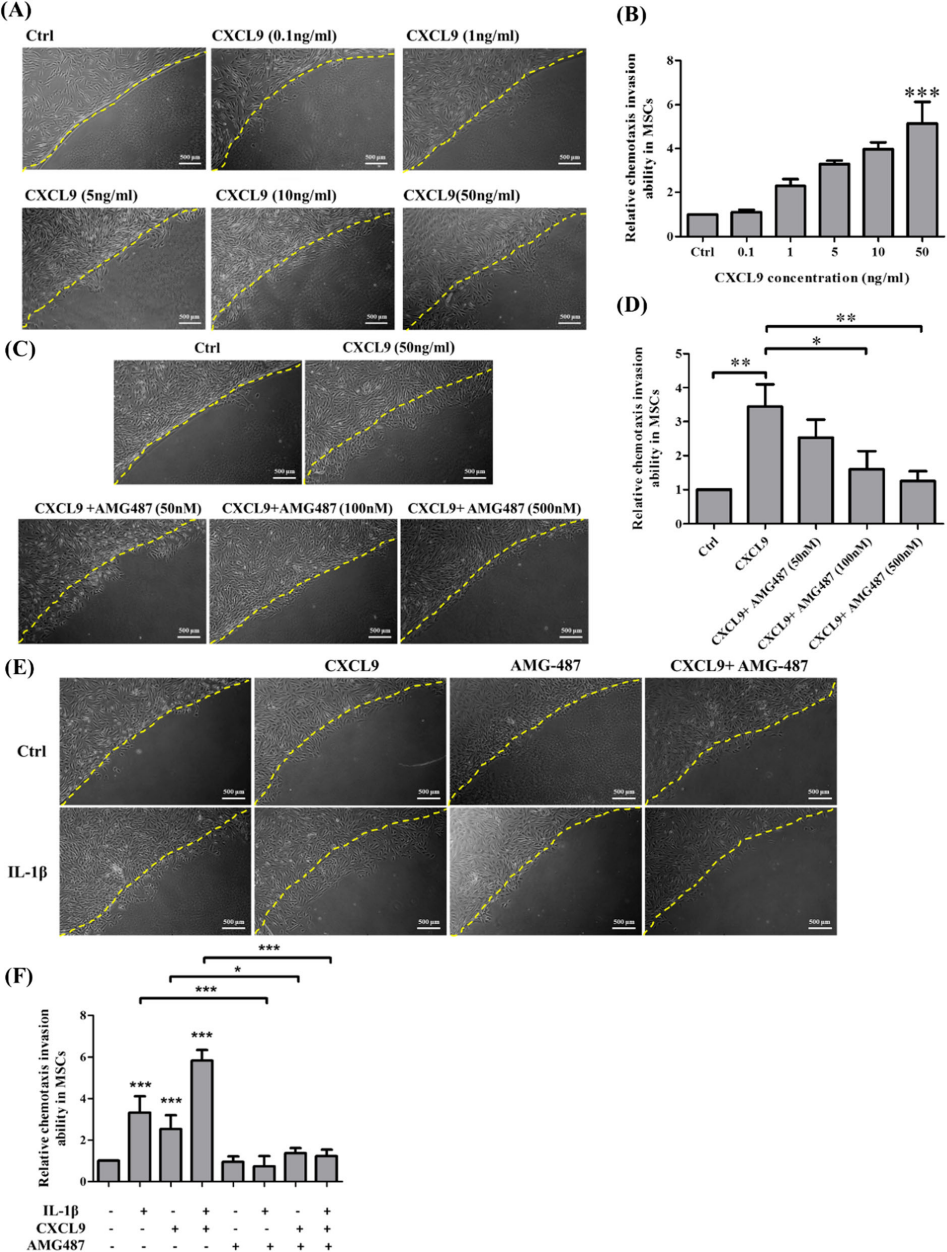
CXCL9 induces MSCs chemotaxis invasion and the effects of CXCR3 antagonist in CXCR3-mediated MSC chemotaxis invasion induced by IL-1β. **a** MSCs were plated on gelatin-coated coverslips in 6-well plates with a 0.5% agarose drop supplemented with different concentrations of CXCL9. After incubation for 24 h, CXCL9 directed chemotaxis of MSCs in agarose drop assay for chemotactic invasion. Scale bars = 500 μm. **b** Quantitative results showing the chemotaxis invasion ability of MSCs migrated under agarose drop. The data represent mean ± SD (*n* = 3). Statistical analysis was determined by Student’s *t* test and one-way ANOVA. ***P < 0.001. **c** MSCs were pretreated with AMG-487 for 2 h and plated on coverslips in 6-well plates with a 0.5% agarose drop supplemented with 50 ng/ml CXCL9. After incubation for 24 h, CXCL9 directed chemotaxis of MSCs in agarose drop assay for chemotactic invasion. Scale bars = 500 μm. **d** Quantitative results showing the chemotaxis invasion ability of MSCs migrating under agarose drop. The data represent mean ± SD (*n* = 3). Statistical analysis was determined by Student’s *t* test and one-way ANOVA. *P < 0.05, ***P < 0.001. **e** MSCs were pretreated or non-pretreated AMG-487 and stimulated with or without IL-1β for 30 min. After pre-activation, MSCs were plated on agarose drop supplemented with the presence or absence of 50 ng/ml CXCL9 for 24 h. Scale bars = 500 μm. **f** Quantitative results showing the chemotaxis invasion ability of MSCs migrated under agarose drop. The data represent mean ± SD (*n* = 3). Statistical analysis was determined by Student’s *t* test and one-way ANOVA. *P < 0.05, ***P < 0.001

It has been found that CXCL9 could enhance the adhering, crawling, and spreading of murine MSCs [19] and enhance cell migration through CXCR3-mediated chemotaxis and transendothelial migration [19, 22]. We examined whether CXCL9 could also induce MSC chemotaxis migration by counting migrated MSCs into agarose drop using agarose drop chemotaxis assay. The results showed that CXCL9 significantly enhanced MSC chemotaxis migration in a dose-dependent manner (Fig. 4a, b). AMG487 is a small molecule antagonist of CXCR3 that can inhibit binding of CXCR3 ligands and cell metastasis. To determine whether AMG484 could inhibit MSC chemotaxis in response to 50 ng/ml CXCL9, MSCs were pretreated with AMG487 for 2 h and migrated MSCs were counted under agarose drop after 24 h CXCL9 induction by agarose drop chemotaxis assay. As shown in Fig. 4c, d, CXCL9 significantly enhanced chemotaxis invasion ability to pass through agarose drop in comparison with the control group. Meanwhile, pretreatment of AMG-487 of MSCs significantly suppressed chemotaxis invasion abilities of MSCs in a dose-dependent inhibition. To further examine the effects of AMG-487 in CXCR3-mediated MSC chemotaxis invasion induced by IL-1β, MSCs were pretreated with AMG-487 for 2 h prior to 30 min IL-1β stimulation. The results showed that IL-1β, CXCL9, or IL-1β plus CXCL9 significantly enhanced chemotaxis invasion ability of MSCs in comparison with the control group, and these effects could be blocked by pretreatment with AMG-487. Moreover, there was no significant difference in pretreatment with AMG-487 alone in comparison to the control group (Fig. 4e, f). The cell viability assay suggests that there was no significant change in AMG487-treated MSCs in comparison to control MSCs. The results show that this concentration of AMG487 did not lead to cell death (Additional file 2: Figure S1B). We thus demonstrated that this antagonist significantly suppressed CXCR3-mediated MSC chemotaxis invasion induced by IL-1β in response to CXCL9.

### CXCR3 antagonist inhibits CXCR3-mediated MSC transendothelial migration induced by IL-1β

**Fig. 5:**
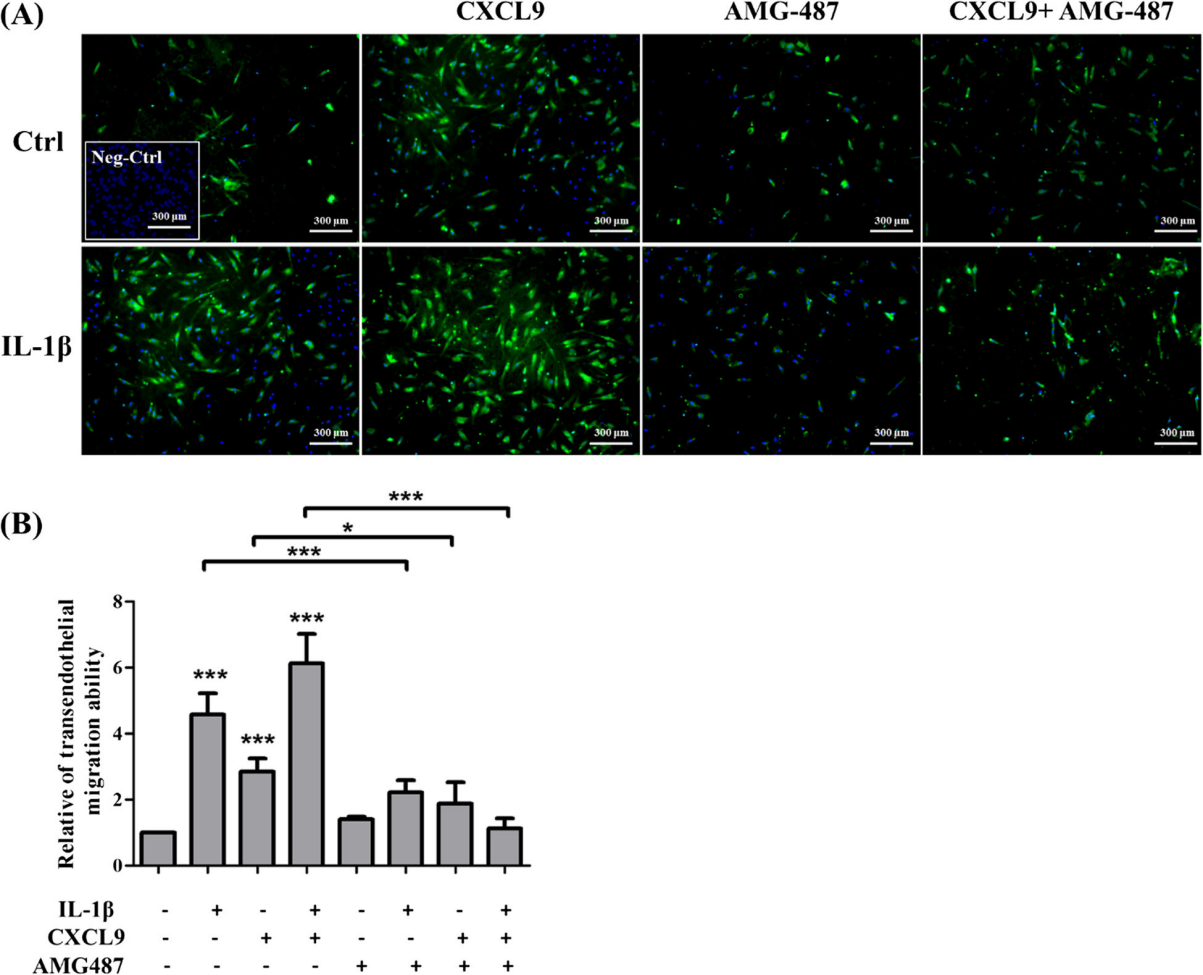
Effects of CXCR3 antagonist in CXCR3-mediated MSC transendothelial migration induced by IL-1β. **a** MSCs were pretreated or non-pretreated AMG-487and stimulated with or without IL-1β for 30 min. After pre-activation, MSCs were plated on activated or non-activated HUVEC monolayer in the upper chamber and incubated in the presence or absence of 50 ng/ml CXCL9 in the lower chamber for 16 h. MSCs: dapi (blue) with cell tracker (green); HUVECs: dapi without cell tracker. Negative control: HUVECs only. Scale bars = 300 μm. **b** Quantitative results showing the transendothelial migration of MSCs to the lower chamber. The data represent mean ± SD (*n* = 3). Statistical analysis was determined by Student’s *t* test and one-way ANOVA. *P < 0.05, ***P < 0.001

In order to assess if MSCs are able to transmigrate through ECs, HUVECs were grown on gelatin-coated membrane of transwells. MSCs were pretreated with AMG-487 for 2 h prior to 30 min of IL-1β stimulation and HUVECs were also treated with IL-1β for 12 h. This study used transendothelial migration assay to investigate the transendothelial migration ability of MSCs from the upper chamber to lower chamber after 16 h incubation. In Fig. 5a, b, IL-1β, CXCL9, or IL-1β plus CXCL9 significantly enhanced the transendothelial migration ability of MSCs in comparison with control MSCs, and these effects could be markedly attenuated by pretreatment with AMG-487. These findings were consistent with our chemotaxis assay. The cell viability assay was performed and indicated no significant change in response to 50 ng/ml CXCL9 in comparison to the control group in both MSCs and HUVECs (Additional file 2: Figure S1C). These results suggested that cell migration was not affected by cell proliferation and that this concentration of CXCL9 did not lead to cell death.

### The p38 MAPK signaling is involved in CXCR3-mediated MSC chemotaxis invasion and MSC transendothelial migration induced by IL-1β

**Fig. 6:**
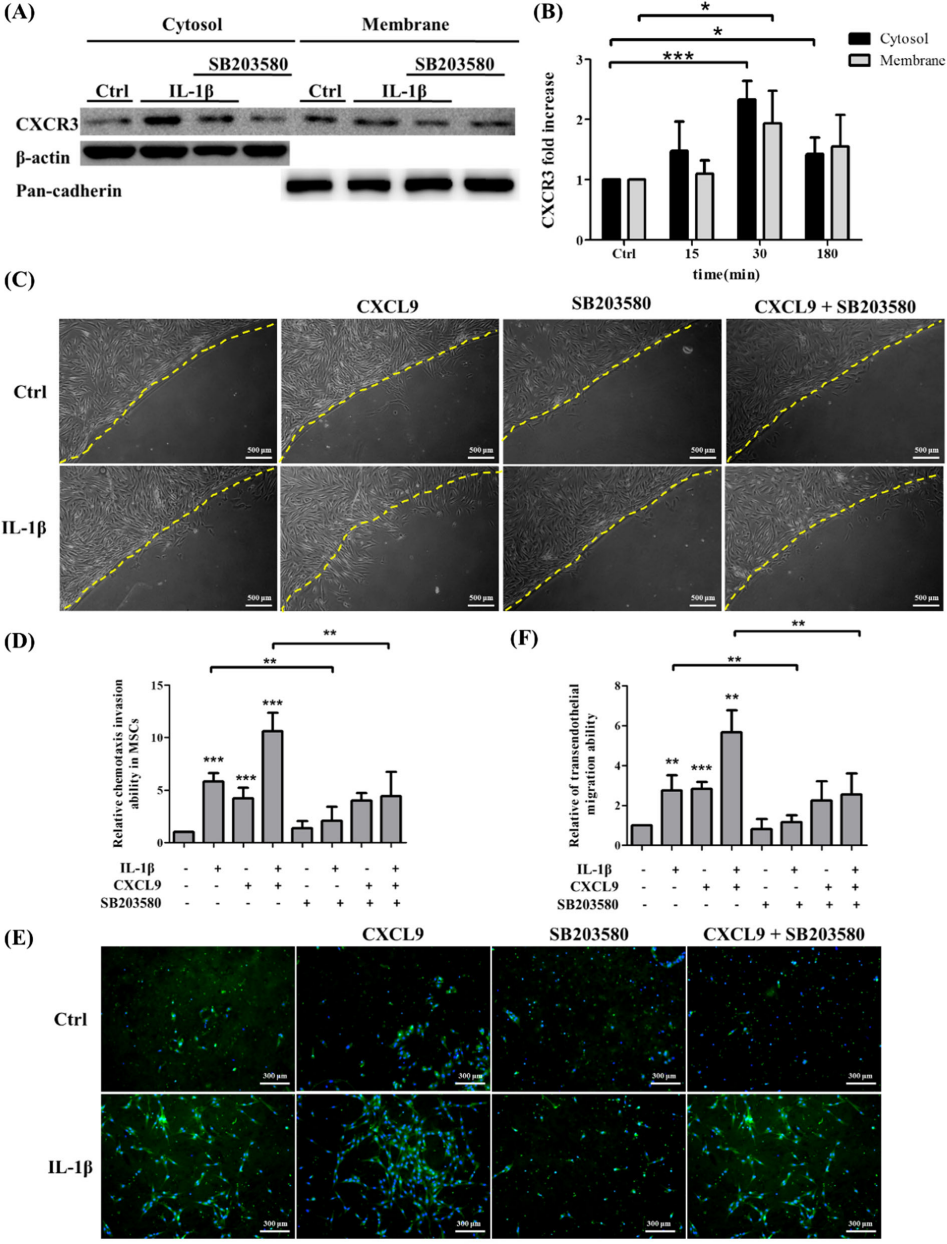
p38 MAPK signaling pathway is involved in CXCR3 protein expression in MSCs, CXCR3-mediated chemotaxis invasion, and transendothelial migration induced by IL-1β. **a** MSCs were pretreated with p38 MAPK inhibitor SB203580, at a concentration of 25 uM, then stimulated with IL-1β for 30 min. The expression of CXCR3 cytosolic and membrane protein were detected by Western blotting. **b** Quantitative results of the Western blotting of CXCR3 cytosolic and membrane protein expression of **a**. The data represent mean ± SD (*n* = 3). Statistical analysis was determined by Student’s *t* test and one-way ANOVA. *P < 0.05, ***P < 0.001. **c** MSCs were pretreated or non-pretreated with SB203580 and stimulated with or without IL-1β for 30 min. After pre-activation, MSCs were plated on agarose drop supplemented with the presence or absence of 50 ng/ml CXCL9 for 24 h. Scale bars = 500 μm. **d** Quantitative results showing the chemotaxis invasion ability of MSCs migrated under agarose drop. The data represent mean ± SD (*n* = 3). Statistical analysis was determined by Student’s *t* test and one-way ANOVA. **P < 0.01, ***P < 0.001. **e** MSCs were pretreated or non-pretreated with p38 MAPK inhibitor and stimulated with or without IL-1β for 30 min. After pre-activation, MSCs were plated on activated or non-activated HUVEC monolayers in the upper chamber and incubated in the presence or absence of 50 ng/ml CXCL9 in the lower chamber for 16 h. Scale bars = 300 μm. **f** Quantitative results showing the transendothelial migration ability of MSCs migrated to the lower chamber. The data represent mean ± SD (*n* = 3). Statistical analysis was determined by Student’s *t* test and one-way ANOVA. **P < 0.01, ***P < 0.001

Previous studies have found that IL-1β induced many kinds of protein expression such as MMP-9 and ICAM-1 through p38 MAPK activation [33, 34]. To investigate whether p38 MAPK signaling was also involved in IL-1β-induced CXCR3 expression on MSCs, MSCs were pretreated with 25 μM p38 MAPK inhibitor SB203580 for 2 h prior to 30 min of IL-1β stimulation. CXCR3 protein expression was analyzed and levels in IL-1β-treated cells were higher than those in non-treated cells. Meanwhile, pretreatment of 25 μM SB203580 of MSCs significantly suppressed IL-1β-induced CXCR3 protein expression both in the cytosol and membrane (Fig. 6a, b). These results confirm that IL-1β induced CXCR3 protein expression on MSCs via the p38 MAPK signaling pathway.

To determine the relevance of p38 MAPK signaling in CXCR3-mediated MSC chemotaxis invasion induced by IL-1β, MSCs were pretreated with p38 MAPK inhibitor SB203580 for 2 h prior to 30 min of IL-1β stimulation. The results showed that IL-1β, CXCL9, or IL-1β plus CXCL9 significantly enhanced chemotaxis invasion abilities of MSCs in comparison with control MSCs. Inhibition of p38 MAPK signaling also significantly attenuated the chemotaxis invasion abilities of IL-1β-treated and IL-1β plus CXCL9-treated MSCs, but did not significantly inhibit the chemotaxis invasion ability of CXCL9-treated MSCs. Moreover, there was no significant difference in pretreatment with SB203580 alone in comparison to the control group (Fig. 6c, d). The cell viability assay showed no significant change in SB203580-treated MSCs in comparison to control MSCs. The results indicate that this concentration of SB203580 did not lead to cell death (Additional file 2: Figure S1A). These studies show that IL-1β upregulated CXCR3 expression on MSCs via p38 MAPK signaling and is also involved in IL-1β-induced MSC chemotaxis invasion. In order to examine the role of p38 MAPK signaling in CXCR3-mediated MSC transendothelial migration induced by IL-1β, MSCs were pretreated with p38 MAPK inhibitor SB203580 for 2 h prior to 30 min of IL-1β stimulation. HUVECs were also treated with IL-1β for 12 h. We then investigated the transendothelial migration abilities of MSCs to transmigrate across HUVECs from the upper chamber to the lower chamber after 16 h. As shown in Fig. 6e, f, treatment with IL-1β and IL-1β plus CXCL9 significantly enhanced transendothelial migration abilities of MSCs in comparison to control MSCs. The effect of transendothelial migration induced by IL-1β and IL-1β plus CXCL9 was attenuated when SB203580 was added to MSCs (Fig. 6e, f). These findings are consistent with our chemotaxis assay. We thus demonstrated that IL-1β-induced MSC CXCR3 expression is mediated by p38 MAPK signaling, then enhances the MSC transendothelial migration.

## Discussion

Mesenchymal stem cells (MSCs) migrate toward injured and inflamed tissue via the bloodstream, possibly attracted by several pro-inflammatory cytokines. Migration and homing involve MSCs attaching to and moving through ECs to the target region to reduce inflammation. During inflammation, ECs upregulate cell surface adhesion molecules and chemoattractants to enhance the transmigration ability of cells [21, 22]. Our previous study has shown that vein-injected MSCs prefer migrating to the pancreas in non-obese diabetic (NOD) mice rather than in normal mice. In addition, higher pancreatic levels of IL-1β were detected in hyperglycemic NOD mice than in normal NOD mice in an ELISA assay [28].

Some studies have shown that IL-1β could induce upregulation of CXC chemokine receptor 3 (CXCR3) in T cells and tumor cells. Previous research reported that CXCR3-specific ligand, CXCL9, a cell chemoattractant expressed in some ECs, can be upregulated in inflammatory microenvironment [21]. Several cell adhesion molecules and chemokines such as ICAM-1 and CXCL5 have been found to be expressed by HUVEC in response to IL-1β stimulation at mRNA levels. However, the expression of chemokine CXCL9 on IL-1β-stimulated HUVEC has not yet been characterized. Our cytokine array data showed that CXCL9 was highly expressed in the pancreas of NOD mice in comparison with normal mice (data not shown). Moreover, we found that MSCs express the CXCR3 chemokine receptor and can be upregulated by adding IL-1β. IL-1β is a pro-inflammatory cytokine and has been associated with inflammation [35] and tissue injury [36]. We then hypothesized that IL-1β-induced expression of CXCL9 in ECs and that IL-1β-induced CXCR3 expression on membrane of MSCs are related to MSC migration toward injured and inflamed tissue via the bloodstream. Therefore, MSCs stimulated with IL-1β prior to local implantation or intravenous delivery would be a better therapeutic strategy.

Our qPCR result indicates that CXCL9 ligands significantly increased (threefold) in IL-1β-treated HUVECs in comparison to non-treated HUVECs. The IL-1β-induced mRNA expression of CXCL9 is further supported by ELISA assay. Expression of CXCL9 ligand in the culture supernatants is 2.5-fold higher than control in IL-1β-treated HUVECs. Our immunofluorescence staining and Western blotting data show that CXCR3 expression is upregulated by IL-1β in both the cytosol and membrane of MSCs. After pretreatment with protein synthesis inhibitor cycloheximide, we found that cycloheximide suppressed IL-1β-induced rapid and transient activation of CXCR3 on cell surface of MSCs in 30 min. In Additional file 3: Figure S2, we also show long-term stimulation of IL-1β in MSCs by immunofluorescence staining. At 6-, 12-, and 24-h periods of time, there was no significant difference in the cytosol and membrane between non-treated and IL-1β-treated MSCs. These results suggest that IL-1β induces CXCR3 expression on cell surface of MSCs via protein synthesis.

It has been observed that co-culture of non-activated MSCs and TNF-α-activated ECs and bone marrow-MSCs showed morphological changes and transmigrated through endothelial monolayers with filopodia [4, 5, 37]. However, there are no studies of in vitro adhesion and migration of co-cultured IL-1β-stimulated ECs and MSCs. Our monolayer co-cultivation assay shows that the time required for human Wharton’s jelly-derived MSCs to attach to HUVECs (within 30 min) is faster than bone marrow-derived MSCs (2 h) [38] under normal conditions. IL-1β stimulation for 30 min significantly facilitates MSC attachment and transendothelial migration efficiency. Non-treated MSCs extended long podia and embed into the edge of HUVECs at 240 min, while IL-1β-treated MSCs transmigrated underneath HUVEC monolayers at the same time.

CXCL9 has been shown to induce chemotaxis in many cells such as melanoma cells [19], T cells [39], and HEK293 cells [40]. In this study, we embed CXCL9 into agarose gel to simulate an inflamed environment. MSCs invade into agarose and migrate under agarose drop in response to CXCL9. We confirmed that CXCL9 significantly enhances MSC chemotaxis migration in a dose-dependent manner by agarose drop assay. Since CXCL9 does not influence cell proliferation and survival (Fig. 5), these results confirm that CXCL9 is a chemoattractant for MSCs. In addition, CXCR3 antagonist AMG-487 significantly suppresses CXCR3-mediated MSC chemotaxis invasion and transendothelial migration induced by IL-1β in response to CXCL9. These results suggest that AMG-487 inhibited binding of CXCL9 and then blocked IL-1β-induced chemotaxis invasion and transendothelial migration. There is no significant difference in pretreatment with AMG-487 alone in comparison to the control group. We consider the low expression of CXCR3 on the surface of MSCs before stimulation. Interestingly, AMG-487 inhibits not only the number of migrated MSCs but also the distance of migrated MSCs (Fig. 4c, f). The p38 MAPK activation was involved in IL-1β-mediated signal transduction [24, 41, 42]. We further examined the effects of the MAPK family (p38, JNK, ERK1/2) and AKT in IL-1β-induced CXCR3 expression in MSCs. MSCs were pretreated with SB203580 (p38 MAPK inhibitor), GSK690693 (AKT inhibitor), SP600125 (JNK inhibitor), and U0126 (ERK1/2 inhibitor) and stimulated with IL-1β for 30 min. The immunofluorescence staining results showed that SB203580 can inhibit the CXCR3 expression in MSCs (Additional file 4: Figure S3). Our Western blotting results indicate that pretreatment with p38 MAPK inhibitor SB203580 suppressed IL-1β-induced CXCR3 protein expression both in the cytosol and membrane. Moreover, SB203580 reduces the chemotaxis invasion and transendothelial migration induced by IL-1β in MSCs. Thus, it appears that IL-1β enhances the level of CXCR3 expression via p38 MAPK signaling pathway to increase the chemotaxis invasion and transendothelial migration ability of MSCs. There is no significant difference in CXCL9 alone compared to CXCL9 plus SB203580. We consider from this that p38 MAPK signaling is not involved in CXCL9 chemotaxis in MSCs.

Local implantation and systemic intravascular administration are the two available methods of delivery in MSC therapy. Due to risks from transplantation and inefficient migration to the target area, the efficacy of MSC therapy has not been well established. In order to resolve these problems, it is important to increase the migration efficiency of MSCs. For MSCs homing to the target region, MSCs need to express a chemokine receptor that recognizes chemokines produced at injured and inflamed tissues. Much is known about related protein CXCR4 expressed on MSCs through SDF-1/CXCR4 axis in its promotion of MSC migration ability [26]. Bone marrow-derived mesenchymal stem cells migrated to the injured margins and contributed to wound repair through CXCL12/CXCR4 signaling [43]. Moreover, these small molecules of chemokines can be upregulated by TNF and IL-1 in an inflammatory environment [44] and affect stem cell and progenitor cell migration and homing abilities. Through chemokine-chemokine receptor axis, homing may provide an important clinical application of MSCs as a cellular vehicle for not only tissue regeneration but also anticancer therapeutics in tumors [45]. In this study, we found that IL-1β upregulated CXCR3 expression on MSC surface and CXCL9 expression in ECs. This effect can increase CXCR3 to CXCL9 binding and promote MSC homing in related diseases.

## Conclusions

**Fig. 7:**
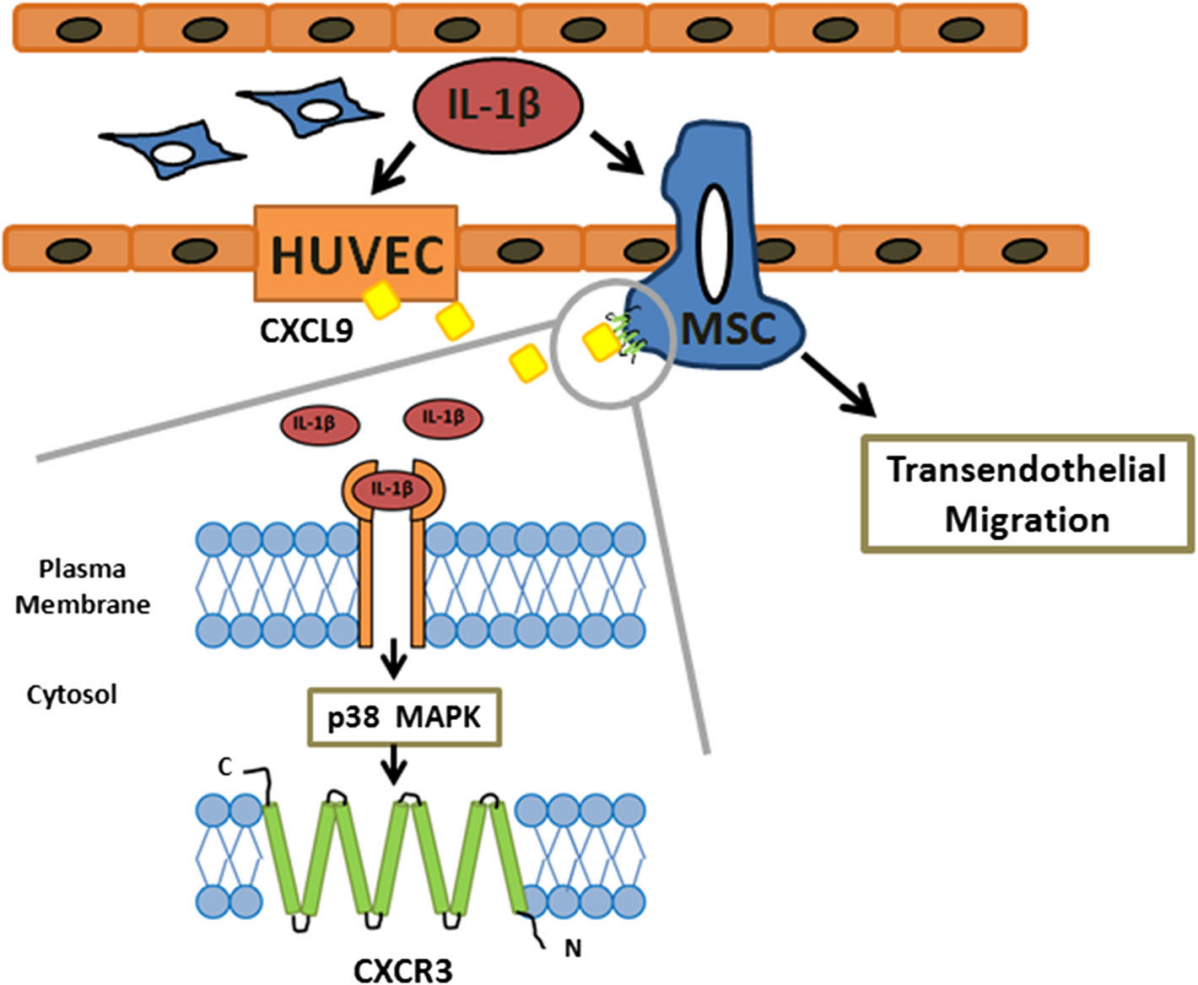
Schematic diagram of IL-1β signaling pathway in CXCR3-mediated MSC transendothelial migration. A schematic diagram depicts the proposed role of IL-1β signaling pathway in MSC transendothelial migration. The process of cell migration is initiated by IL-1β through p38 MAPK-induced expression of CXCR3 in MSCs in response to CXCL9

In this study, we demonstrated that IL-1β induced CXCR3 expression via p38 MAPK signaling and further increased CXCR3-CXCL9 axis signaling in MSC chemotaxis invasion and transendothelial migration (Fig. 7). The findings of the study hold significance for the future development of MSC therapies targeting inflammatory diseases. Stimulation of MSCs with IL-1β prior to local implantation or intravenous delivery would be one promising potential therapeutic strategy.

## Additional files

Additional file 1: **Table S1.** Sequences of primers used for quantitative real-time polymerase chain reaction experiments. (DOCX 12 kb)

Additional file 2: **Figure S1.** Cell viability of MSCs, HUVECs treated with IL-1β, inhibitors, and CXCL9. (A) Cell viability assay for IL-1β and inhibitor-treated MSCs in this research. Results were quantified using multimode microplate readers at a wavelength of 545 nm. The data represent mean ± SD (*n* = 3). (B) Cell viability assay for IL-1β-treated HUVECs. Results were quantified by multimode microplate readers at a wavelength of 545 nm. The data represent mean ± SD (*n* = 3). (C) Cell viability assay for CXCL9-treated in MSCs and HUVECs. Results were quantified by multimode microplate readers at a wavelength of 545 nm. The data represent mean ± SD (*n* = 3). Statistical analysis was determined by Student’s *t* test and one-way ANOVA. (DOCX 156 kb)

Additional file 3: **Figure S2.** Short-term and long-term stimulation of IL-1β in MSCs. (A) Immunofluorescence staining of CXCR3 (green) in control MSCs stimulated with IL-1β at 15, 30, and 180 min; cellular nuclei were stained in blue. Scale bar: 50 μm. (B) MSCs stimulated with IL-1β at 6, 12, and 24 h. Scale bar: 50 μm. (DOCX 912 kb)

Additional file 4: **Figure S3.** Effects of MAPK Family (p38, JNK, ERK1/2) and AKT in IL-1β-induced CXCR3 expression in MSCs. Immunofluorescence staining of CXCR3 expression on MSCs. MSCs were pretreated with SB203580 (p38 MAPK inhibitor), GSK690693 (AKT inhibitor), SP600125 (JNK inhibitor), and U0126 (ERK1/2 inhibitor) and stimulated with IL-1β for 30 min. Scale bar: 50 μm. (DOCX 1219 kb)

## Abbreviations

MSCs: Mesenchymal stem cells
ECs: Endothelial cells
HUVECs: Human umbilical vein endothelial cells
IL-1β: Interleukin-1β
CXCL9: Chemokine (C-X-C motif) ligand 9
CXCR3: CXC chemokine receptor 3
TGF-β1: Transforming growth factor-β1
SDF-1: Stromal cell-derived factor 1
TNF-α: Tumor necrosis factor-α
