# Supplementary material for: Interleukin-1β induces CXCR3-mediated chemotaxis to promote umbilical cord mesenchymal stem cell transendothelial migration
Source: Stem Cell Res Ther. 2018 Oct 25;9:281. doi: 10.1186/s13287-018-1032-9 (PMC6202827; doi:10.1186/s13287-018-1032-9)
Supplement: Supplementary file 1 — Table S1. Sequences of primers used for quantitative real-time polymerase chain reaction experiments. (DOCX 12 kb) [file 13287_2018_1032_MOESM1_ESM.docx]

Table S1. Sequences of primers used for Quantitative Real-Time Polymerase Chain Reaction experiments.

| CXCL9 forward primer | 5’- AGTGCAAGGAACCCCAGTAG-3’ |
| --- | --- |
| CXCL9 reverse primer | 5’-GTGGATAGTCCCTTGGTTGGT-3’ |
| GAPDH forward primer | 5’-GAAGGTGAAGGTCGGAGTCAAC-3’ |
| GAPDH reverse primer | 5’-CAGAGTTAAAAGCAGCCCTGGT-3’ |
