# Supplementary figures and images for: Interleukin-1β induces CXCR3-mediated chemotaxis to promote umbilical cord mesenchymal stem cell transendothelial migration
Source: Stem Cell Res Ther. 2018 Oct 25;9:281. doi: 10.1186/s13287-018-1032-9 (PMC6202827; doi:10.1186/s13287-018-1032-9)

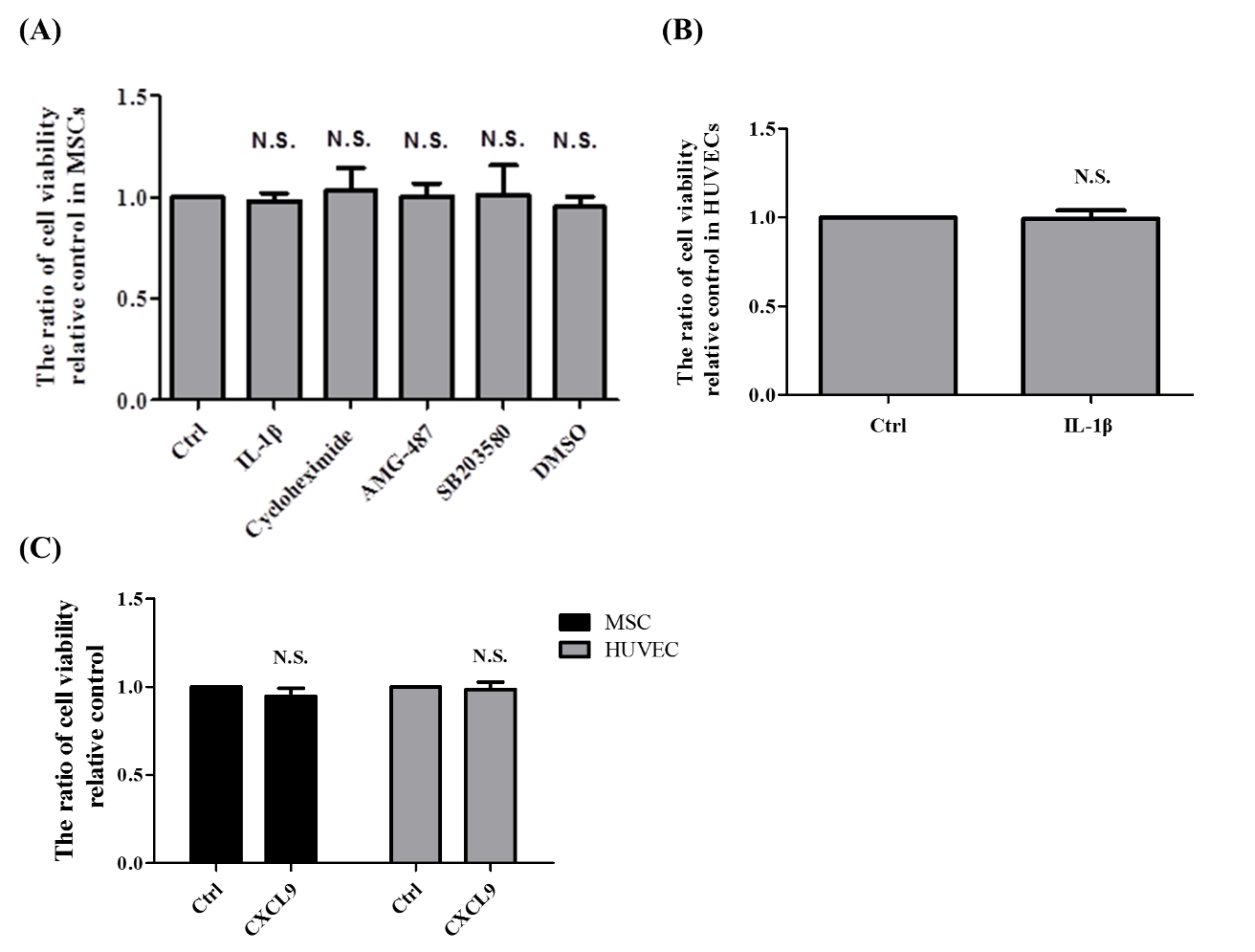

Supplement: Supplementary file 2 — Figure S1. Cell viability of MSCs, HUVECs treated with IL-1β, inhibitors, and CXCL9. (A) Cell viability assay for IL-1β and inhibitor-treated MSCs in this research. Results were quantified using multimode microplate readers at a wavelength of 545 nm. The data represent mean ± SD (n = 3). (B) Cell viability assay for IL-1β-treated HUVECs. Results were quantified by multimode microplate readers at a wavelength of 545 nm. The data represent mean ± SD (n = 3). (C) Cell viability assay for CXCL9-treated in MSCs and HUVECs. Results were quantified by multimode microplate readers at a wavelength of 545 nm. The data represent mean ± SD (n = 3). Statistical analysis was determined by Student’s t test and one-way ANOVA. (DOCX 156 kb) [file 13287_2018_1032_MOESM2_ESM.docx]

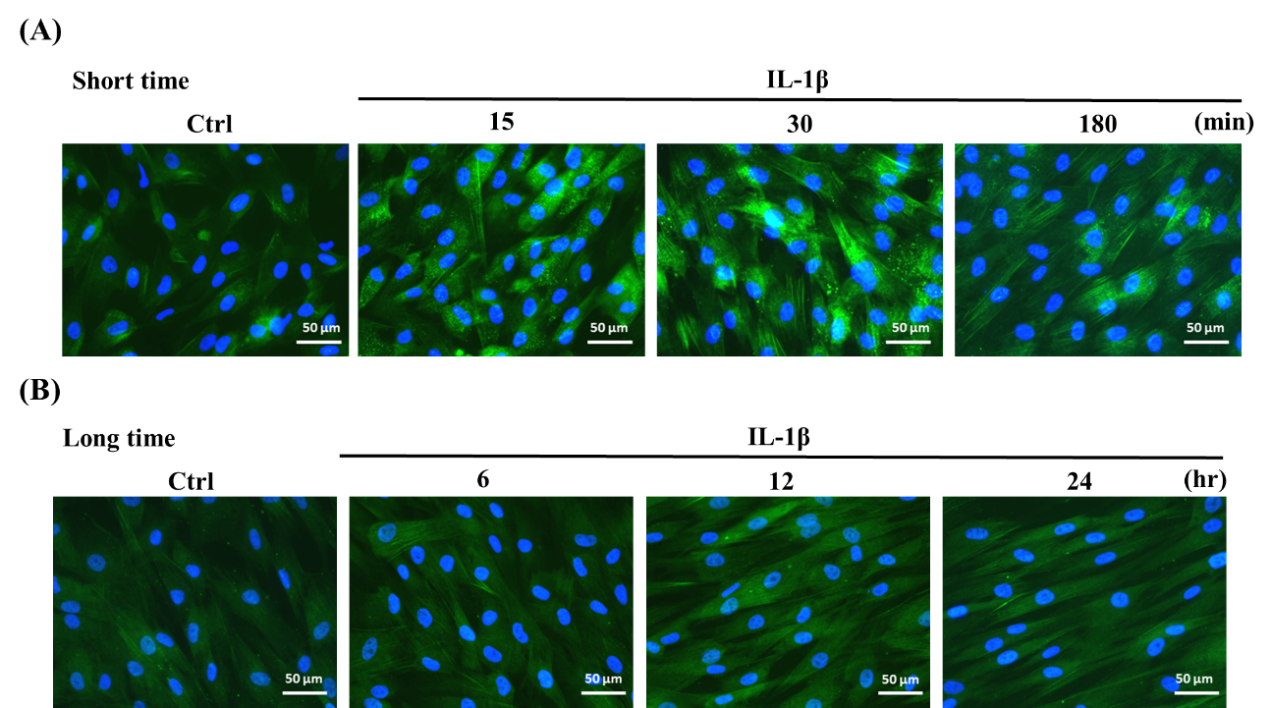

Supplement: Supplementary file 3 — Figure S2. Short-term and long-term stimulation of IL-1β in MSCs. (A) Immunofluorescence staining of CXCR3 (green) in control MSCs stimulated with IL-1β at 15, 30, and 180 min; cellular nuclei were stained in blue. Scale bar: 50 μm. (B) MSCs stimulated with IL-1β at 6, 12, and 24 h. Scale bar: 50 μm. (DOCX 912 kb) [file 13287_2018_1032_MOESM3_ESM.docx]

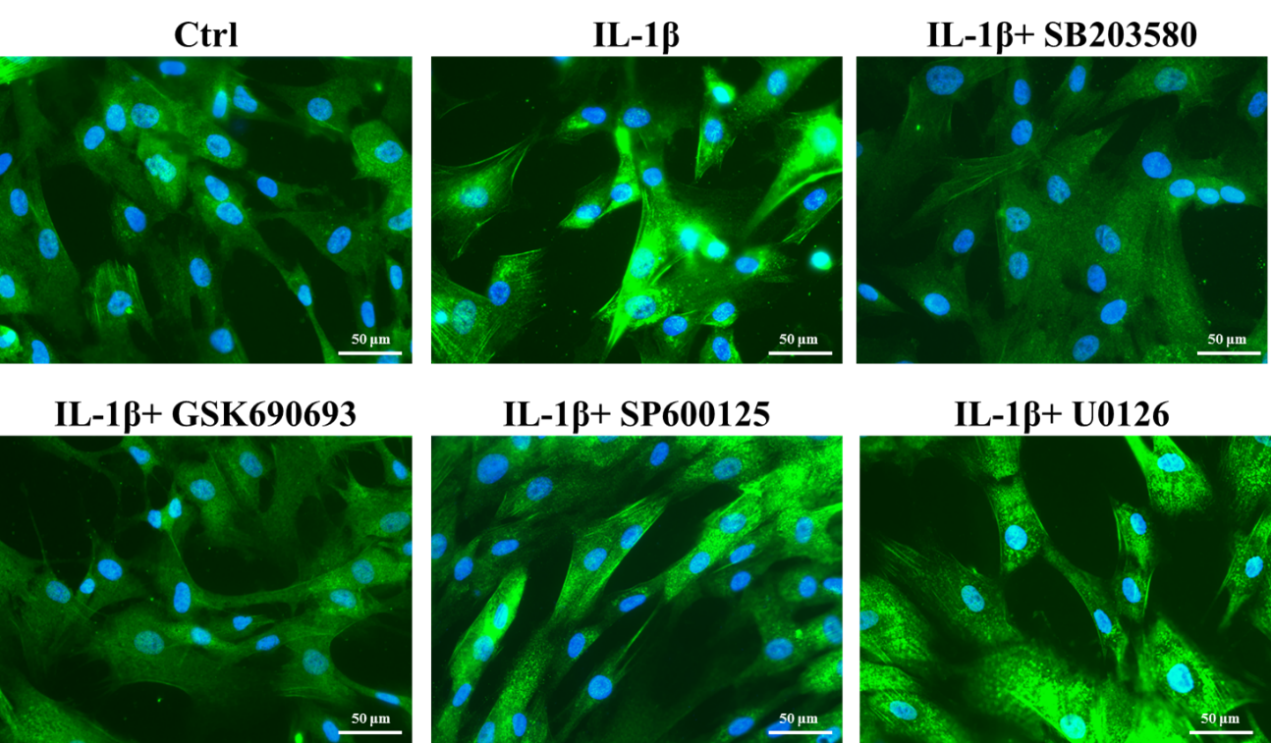

Supplement: Supplementary file 4 — Figure S3. Effects of MAPK Family (p38, JNK, ERK1/2) and AKT in IL-1β-induced CXCR3 expression in MSCs. Immunofluorescence staining of CXCR3 expression on MSCs. MSCs were pretreated with SB203580 (p38 MAPK inhibitor), GSK690693 (AKT inhibitor), SP600125 (JNK inhibitor), and U0126 (ERK1/2 inhibitor) and stimulated with IL-1β for 30 min. Scale bar: 50 μm. (DOCX 1219 kb) [file 13287_2018_1032_MOESM4_ESM.docx]
